# Cyclin L1 participates in Adriamycin resistance and progression of osteosarcoma via PI3K/AKT-mTOR pathway

**DOI:** 10.18632/aging.205972

**Published:** 2024-06-26

**Authors:** Yanbin Zhang, Tao Zhang, Long Chen, Zijun Guo, Xiaobing Jiang

**Affiliations:** 1 Department of Neurosurgery, Union Hospital, Tongji Medical College, Huazhong University of Science and Technology, Wuhan 430022, China; 2 Department of Gastroenterology, Zhongnan Hospital of Wuhan University, Wuhan 430062, China; 3 Department of Anesthesiology, Union Hospital, Tongji Medical College, Huazhong University of Science and Technology, Wuhan 430022, China; 4 Key Laboratory of Anesthesiology and Resuscitation, Huazhong University of Science and Technology, Ministry of Education, Wuhan 430022, China; 5 Institute of Anesthesia and Critical Care Medicine, Union Hospital, Tongji Medical College, Huazhong University of Science and Technology, Wuhan 430022, China

**Keywords:** osteosarcoma, CCNL1, chemoresistance, PI3K/AKT-mTOR, Adriamycin, prognosis

## Abstract

Chemoresistance is a common and thorny problem in the treatment of osteosarcoma (OS), which obstructs the response of relapse or metastasis of OS to chemotherapy and leads to the unfavorable prognosis of OS patients. Cyclin L1 (CCNL1) is a non-canonical cyclin that plays an important role in the regulation of tumor cell proliferation and lymph node metastasis. In this work, we explored the impact of CCNL1 expression levels on proliferation, migration, and Adriamycin (ADM) resistance in OS and related mechanisms. We found that CCNL1 expression levels were significantly associated with clinical prognosis of patients with OS and CCNL1 could promote OS proliferation and migration. In addition, we also revealed that cellular CCNL1 was significantly increased in ADM-resistant OS cells and promoted ADM resistance. The PI3K/AKT-mTOR pathway is involved in CCNL1-mediated ADM resistance in OS. In summary, CCNL1 is involved in the progression of ADM resistance and OS through the PI3K/AKT-mTOR pathway, which will provide a new clue to the mechanism of ADM resistance and a potential target for the treatment of ADM-resistant OS.

## INTRODUCTION

OS is a common primary bone malignancy, especially in children and adolescents [1]. The tendency of OS to metastasize and spread makes it difficult for conventional surgical resection to achieve a curative effect [2]. The introduction of MAP chemotherapy regimens (including Adriamycin, methotrexate, and cisplatin) has significantly improved the prognosis of OS [3, 4]. Nonetheless, as one of the main causes of treatment failure, the occurrence of chemoresistance is still a problem that needs to be solved urgently [5, 6]. Therefore, there is an urgent need to clarify the mechanisms of chemoresistance in OS and translate it into clinical treatment.

CCNL1 is not only a cell cycle regulatory protein, but also a potential oncogene, which can regulate tumor cell proliferation, invasion, drug resistance and other tumor-related biological behaviors [7]. Previous reports have shown that CCNL1 is involved in regulating the occurrence and development of various tumors, such as head and neck cell carcinoma [7, 8], prostate cancer [9], pancreatic cancer [10], EBV-positive nasopharyngeal carcinoma [11] and uterine cervical carcinoma [12]. However, the role and related mechanisms of CCNL1 in the development and drug resistance of OS remain unclear.

In this work, we found that CCNL1 was associated with Huvos grade and prognosis of OS patients. Then, a series of experiments were conducted to explore and verify the role and related mechanisms of CCNL1 in the process of ADM-resistance of OS. In summary, we found that CCNL1 participates in ADM resistance and progression of OS via PI3K/AKT-mTOR pathway, which not only provides a new clue to the mechanism of ADM resistance in OS, but also provides a potential clinical option for its treatment.

## MATERIALS AND METHODS

### Data source and survival analysis

The OS tissue gene expression profiles and the related clinical information of GSE21257 (*n* = 53), and GSE39058 (*n* = 42) were downloaded from GEO database (http://www.ncbi.nlm.nih.gov/geo). Another OS cohort containing RNA-seq data and the related clinical information (*n* = 88) was downloaded from TARGET database (https://ocg.cancer.gov/programs/target). The clinical information of an OS cohort GSE39040 (*n* = 65) was obtained from GEO database. Kaplan–Meier log-rank test, uni- and multivariate Cox regression analyses were used to analyze the prognostic value of Huvos grade and CCNL1.

### Cell and reagents

hFOB, HOS and 143B were obtained from the China Center (Wuhan, China) for Type Culture Collection. ADM-resistant cell HOS/ADM and 143B/ADM were acquired by sequential exposure to increasing doses of ADM. The OS cells were cultured in α-MEM medium (HyClone, UT, USA) with 10% fetal bovine serum (FBS) (Gibco, NY, USA) and 1% penicillin-streptomycin at 37°C with 5% CO_2_. The chemotherapy drugs (Adriamycin, Methotrexate, and Cisplatin) were purchased from Solarbio (Beijing, China).

### Transfection

The lentiviruses against CCNL1 (Lv-shCCNL1) were purchased from GeneChem (Shanghai, China). In addition, the pcDNA3.1 vector containing the CCNL1 cDNA sequence was used to overexpress CCNL1. Furthermore, 2 to 4-week puromycin at 2.5 g/ml (Sigma-Aldrich, MO, USA) administration was used to select single-cell clones following lentiviral infection.

### Quantitative real-time PCR (qRT-PCR)

Total RNA was isolated using Trizol reagent (Invitrogen, MA, USA) from HOS and 143B cells and measured by qRT-PCR. 1 μg of total RNA was reverse transcribed into reverse transcription (cDNA) using Prime Script RT Master Mix (Vazyme, Nanjing, China). QRT-PCR was performed using the LightCycler^®^ 480 SYBR I Master Mix (Roche, Switzerland). GAPDH was used for internal control. The primers used for qRT-PCR can be seen on Supplementary Table 1.

### Western blot

All proteins were isolated using a protein extraction kit (Beyotime, Shanghai, China). After separating proteins by SDS-PAGE electrophoresis, the proteins were transferred to polyvinylidene fluoride (PVDF) membranes (Millipore, MA, USA) and blocked with 5% skimmed milk for two hours. Then, membranes were incubated with antibodies against MRP1 (Cell Signaling Technology, USA, CST-72202), P-gp (Cell Signaling Technology, USA, CST-13342), Survivin (Cell Signaling Technology, USA, CST-2808), AKT (Abcam, USA, ab8805), p-AKT (Abcam, USA, ab38449), mTOR (Cell Signaling Technology, USA, CST-2983), p-mTOR (Cell Signaling Technology, USA, CST-5536), MMP2 (Proteintech, USA, 10373-2-AP), CCNL1 (Abcam, USA, ab108935) and GAPDH (Abcam, USA, ab8245) overnight at 4°C. The membranes were then rinsed three times with TBST in room temperature, and the secondary antibody (goat anti-rabbit, 1:1000) was added. Protein bands with the ECL Substrate (Thermo Fisher Scientific, MA, USA) were visualized by the FluorChem Imaging System (ProteinSimple, CA, USA).

### MTT assay

HOS and 143B cells in the exponential growth phase were seeded into a 96-well plate by 2000 cells per well. Then the cells were incubated for 4 h with 20 μl of MTT solution (Servicebio, Wuhan, China). A Microplate Reader (Biotech Instruments μQuant, USA) was utilized to test the absorbance values at 490 nm. At least five replicates were used in each MTT experiment, which was repeated three times. The resistance index (RI) of this study was calculated by the ratio of the half inhibitory concentration of ADM-resistant OS cells to their parental cells.

### Transwell assay

To measure cell migration capacity, cells in 0.2 ml serum-free α-MEM medium were seeded in the apical chamber of each Transwell chamber, while the basolateral chamber was filled with 0.6 ml DMEM supplemented with 10% FBS. After 24 h incubating, the migrated cells in the lower parts were stained by 0.1% crystal violet dye (Servicebio, Wuhan, China) for 20 min after fixed with 4% paraformaldehyde for 10 min. The migrated cells were recorded and calculated by using the ImageJ software.

### Wound healing assay

OS cells were seeded in 6-well plates and cultured to confluence, and then the OS cell monolayer was scraped using a 10 μL pipette tip. After washing with PBS, the wound areas were photographed under a microscope at 0 and 24 h after scratching. OS cell mobility was measured by a caliper and used the following formula to define the percentage of the repaired area. Calculation method: (1-(current size/initial wound size)) × 100%.

### TUNEL staining

The OS cells were soaked in paraformaldehyde (4%) for 1 hour and then incubated with 3% H_2_O_2_ and 0.1% Triton X-100 for 10 min. The cells were co-stained with TUNEL inspection solution and DAPI (stains nuclei). Afterward, three slides were randomly selected and visualized by a fluorescence microscope (Olympus, Tokyo, Japan). Apoptotic index formula: (number of apoptotic cells/total cells).

### Xenograft assays

All studies were approved by the medical ethical committee and conducted according to the guidelines of the Centre of Experimental Animal Tongji Medical College of Huazhong University of Science and Technology (Wuhan, China). Specific-pathogen-free male nude mice aged 4–5 wk and weighed around 20 g were purchased from Vitalriver (China). HOS/ADM cells transferred with sh-NC control or sh-CCNL1 in 200 μL saline (3 × 10^6^) were injected into the mice. A total of 24 nude mice with ~100-mm^3^ tumors were divided into 4 groups randomly. The tumor volume and body weight of nude mice were measured every 4 days. The animal experiments were terminated on day 28 and the mice were euthanized. The tumor volume calculation formula: length × width^2^/2.

### Gene set enrichment analysis (GSEA)

GSEA was performed to determine the significantly enriched biological processes or pathways in OS patients with low and high levels of CCNL1 expression using the ‘Clusterprofiler’ R package [13] based on the Kyoto Encyclopedia of Genes and Genomes (KEGG) database in GSE39058.

### Statistical analyses

All results were representative of at least three independent experiments and all data were given as mean ± SD. Two-sided student’s *t*-tests or analysis of variance (ANOVA) tests were used to compare two or more than two groups with normally distributed variables, respectively. Chi-square test for cohort correlation analysis was used. Log-Rank test was used for survival analysis. Statistically significant differences were considered when *p* < 0.05.

### Availability of data and materials

All data generated or analyzed during this study are available from the corresponding author on reasonable request.

## RESULTS

### The significant prognostic value of Huvos grade in OS

**Figure 1:**
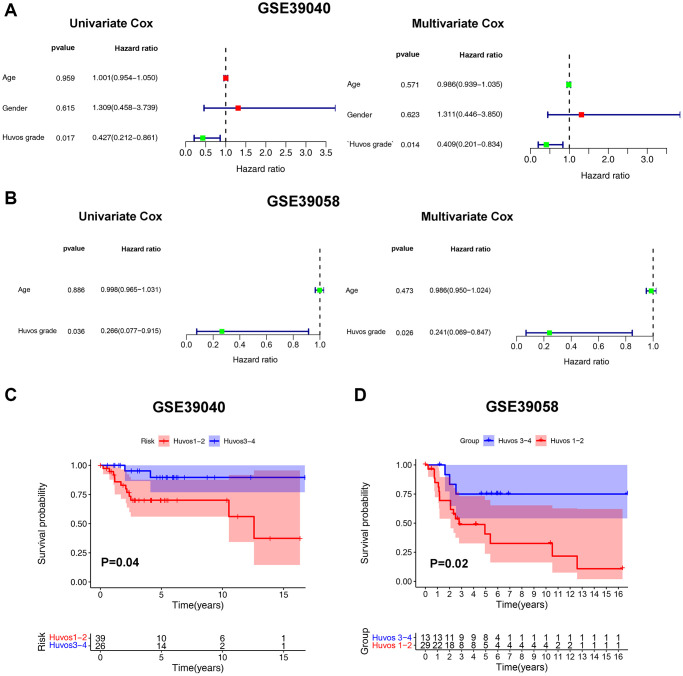
**The Huvos grades - an independent risk factor for OS overall survival.** The Cox regression analysis of Huvos grade in GSE39040 (**A**) and GSE39058 (**B**). The overall survival analysis between Huvos 1–2 and Huvos 3–4 Huvos grades in GSE39040 (**C**) and GSE39058 (**D**).

Univariate and multivariate Cox regression analysis results of the GSE39040 showed that Huvos grade was an independent risk factor for the prognosis of OS patients (Figure 1A). Moreover, the results were validated in the GSE39058 (Figure 1B) and GSE21257 cohorts (Supplementary Figure 1A). The Kaplan-Meier survival analysis results showed that lower Huvos grade (Huvos 1–2) OS had a significantly unfavorable prognosis in GSE39040 and GSE39058 (Figure 1C, 1D), but not in GSE21257 (Supplementary Figure 1B).

### The significant prognostic value of CCNL1 in OS

**Figure 2:**
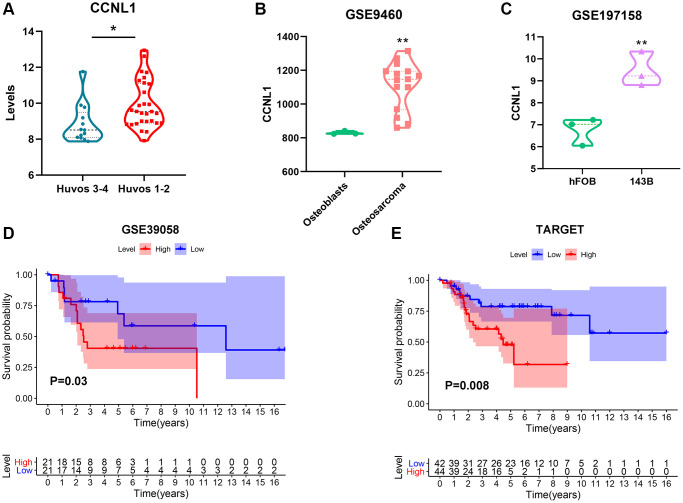
**The expression and prognostic value of CCNL1 in OS.** The expression of CCNL1 was overexpressed in low (Huvos 1–2) Huvos grade OS patients ^*^P < 0.05 vs. Huvos 3–4 (**A**). CCNL1 level was higher in OS tissue ^**^P < 0.01, vs. Osteoblasts (**B**), and cell lines ^**^P < 0.01, vs. hFOB (**C**). High CCNL1 level was correlated with worse prognosis in OS in GSE39058 and TARGET cohorts (**D**, **E**).

The expression of CCNL1 was higher in low Huvos grade (Huvos 1–2) patients (Figure 2A). Moreover, CCNL1 level was higher in OS tissue (Figure 2B), and cell lines (Figure 2C). Furthermore, the survival analysis showed that high expression of CCNL1 was associated with worse OS prognosis in GSE39058, TARGET, and GSE21257 cohorts (Figure 2D, 2E and Supplementary Figure 1C). Table 1 showed the correlation between the expression level of CCNL1 and OS clinicopathological characteristics in the GSE39058 cohort.

**Table 1:** Correlations between the expression level of CCNL1 with clinicopathological characteristics of OS in GSE39058 cohort.

**Parameters**	**GSE39058**			
	**Low**	**High**	**Total**	***P***
Age (y)				0.469
<16	17	15	32	
>16	4	6	10	
Gender				0.287
Male	9	13	22	
Female	12	9	21	
Huvos grade				0.045
1–2	11	18	29	
3–4	10	3	13	
Recurrence				0.064
Positive	13	7	20	
Negative	8	14	22	

### CCNL1 promoted the viability, proliferation and migration

**Figure 3:**
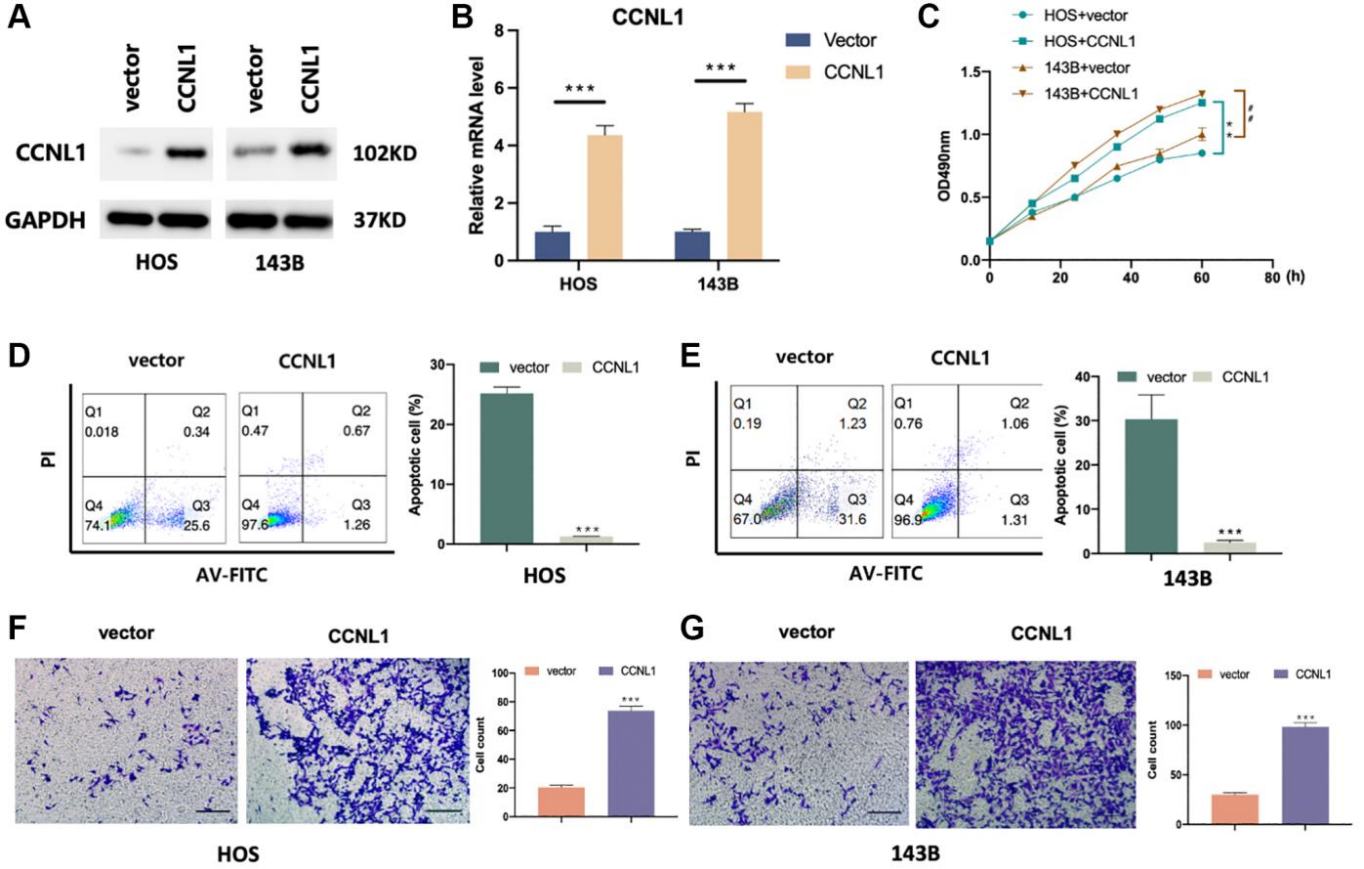
**CCNL1 promotes migration and inhibits apoptosis of OS cells.** CCNL1 expression level in HOS and 143B was examined through western blot (**A**) and qRT-PCR. ^***^P < 0.001 vs. vector (**B**). (**C**) The proliferation of control and CCNL1-overexpressed cells examined through MTT. ^**^P < 0.01, vs. HOS plus vector; ^##^*P* < 0.01, vs. 143B plus vector. (**D**, **E**) The apoptosis rate of CCNL1-overexpressed HOS, 143B cells and the control vector group was analyzed by flow cytometry. ^***^P < 0.001, vs. control. (**F**, **G**) Transwell results of migration ability of HOS and 143B cells. ^***^P < 0.001. (Bar = 200 μm).

We observed that CCNL1 was more expressed in HOS and 143B cells compared with hFOB (Supplementary Figure 2). For exploring the role of CCNL1 in OS, the level of CCNL1 was upregulated in HOS and 143B cells (Figure 3A, 3B). Compared with the control vector group, CCNL1 overexpression was able to promote the OS cells proliferation (Figure 3C). Moreover, as shown in Figure 3D, 3E, CCNL1 overexpression reduced ADM-induced apoptosis (HOS-vector, 30.20% vs. HOS-CCNL1, 1.32%; 143B-vector, 25.20% vs. 143B-CCNL1, 1.25%). Furthermore, as shown in Figure 3F, 3G, the results of Transwell assay suggested that overexpression CCNL1 was able to promote the OS cells migration (HOS-vector, 20.33 vs. HOS-CCNL1, 73.66; 143B-vector, 30.00 vs. 143B-CCNL1, 98.33).

### CCNL1 was associated with ADM resistance

**Figure 4:**
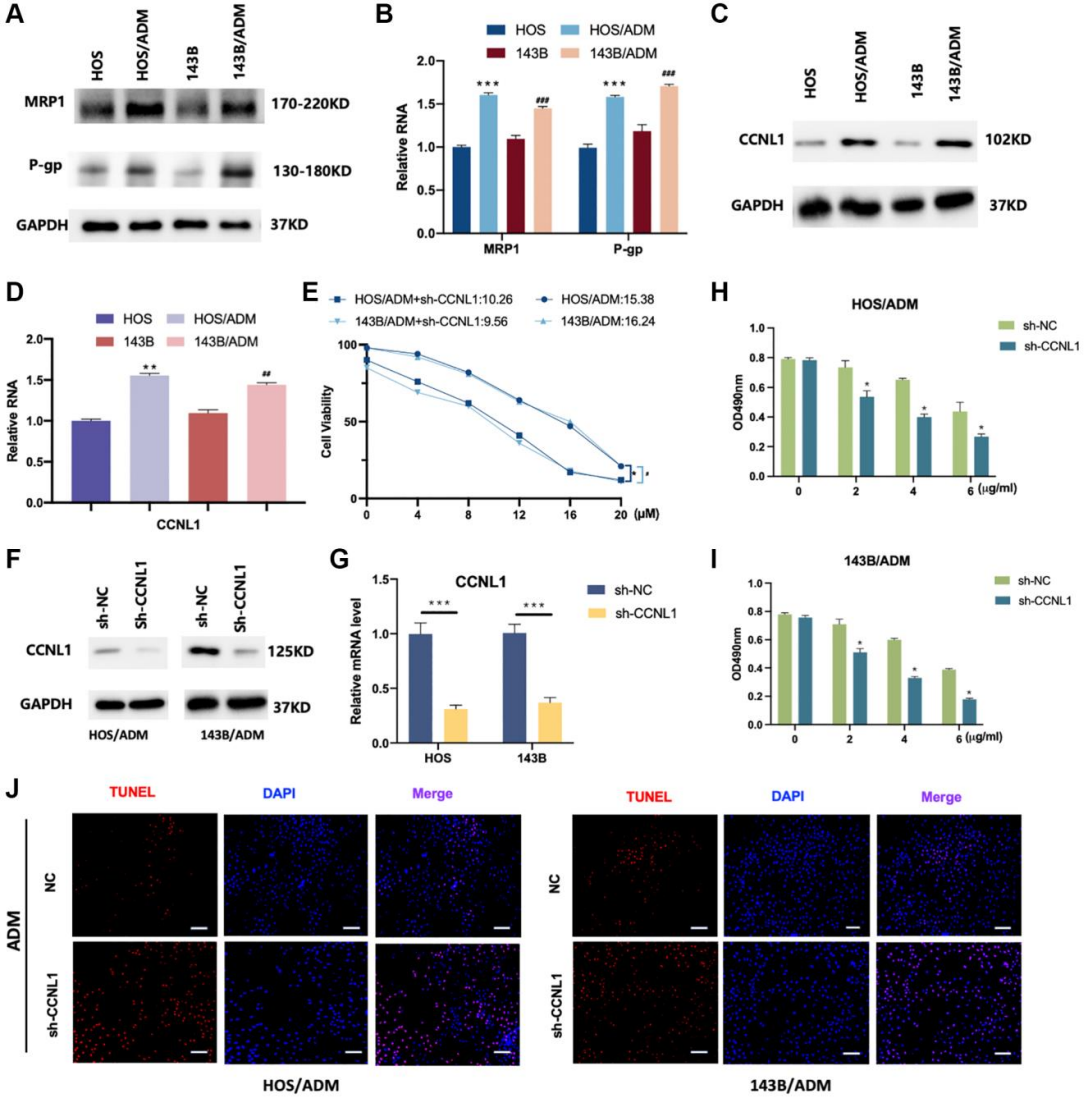
**CCNL1 was associated with ADM resistance.** The expression of MRP1 and P-gp was examined by western blot (**A**) and qRT-PCR analysis (**B**). ^**^P < 0.01, vs. HOS; ^##^*P* < 0.01, vs. 143B. (**C**) The expression level of CCNL1 was examined by western blot and qRT-PCR (**D**). ^**^P < 0.01, vs. HOS; ^##^*P* < 0.01, vs. 143B. (**E**) The viability of the os cells was accessed by MTT assay. ^*^P < 0.05, ^#^*P* < 0.05. The expression of CCNL1 was examined by western blot (**F**) and qRT-PCR analysis (**G**). ^***^P < 0.001, ch-NC vs. sh-CCNL1. The viability of HOS/ADM (**H**) and 143B/ADM cells (**I**) was accessed by MTT assay. The rate of tunnel-positive cells increased in the sh-CCNL1 group in comparison to the NC group, bar = 200 μm (**J**).

The RI and IC_50_ of HOS, HOS/ADM, 143B and 143B/ADM cells were shown in Table 2. The RI values of HOS/ADM and 143B/ADM increased by 19- and 22-fold respectively compared with their parental cells (Table 2). Furthermore, the expression levels of P-glycoprotein (P-gp) and multidrug resistance-associated protein-1 (MRP1) in ADM resistant OS cells were significantly higher than their corresponding parental cells (Figure 4A, 4B).

**Table 2:** The IC_50_ and RI of the OS cells against ADM.

**Cell line**	**IC_50_**	**RI**
HOS	0.78 + 0.02	1
HOS/ADM	15.38 + 0.34	19.71
143B	0.71 + 0.04	1
143B/ADM	16.24 + 0.57	22.87

Unit: μmol/L. IC_50_: the half effective inhibition concentrations. Abbreviation: RI: resistance index.

Additionally, cross-resistance in ADM-resistant OS cells was also explored. The MTT assay was used to determine multidrug resistance in ADM-resistant OS cells exposed to methotrexate (MTX) and cisplatin (DDP). At the IC_50_ level, the RI values of HOS/ADM for MTX and DDP were 7.13 and 3.01 times higher than those of the parental cell lines, respectively. Likewise, the RI values of 143B/ADM against MTX and DDP are 6.54 and 2.25 times higher than that of the parental cell lines, respectively (Table 3), and these resistance assay results showed that ADM resistant cells acquired multidrug resistance (MDR). In summary, the above results indicated the successful establishment of ADM-resistant HOS and 143B cells.

**Table 3:** IC_50_ and RI of the OS cells against MTX or DDP.

	**IC_50_**				**RI**	
	**HOS**	**HOS/ADM**	**143B**	**I43B/ADM**	**HOS/ADM**	**143B/ADM**
MIX	1.85 ± 0.25	13.19 ± 2.21	2.58 ± 0.21	16.88 ± 1.56	7.13	6.54
DDP	3.68 ± 0.34	11.09 ± 1.66	8.11 ± 0.52	18.23 ± 0.28	3.01	2.25

Unit: μmol/L.

For investigating the relationship between CCNL1 and ADM-resistance, the expression level of CCNL1 in ADM-resistant OS cells was detected. As shown in Figure 4C, 4D, the western blot and PCR results demonstrated that the CCNL1 in ADM resistant OS cells were higher than the parental cells. Moreover, as shown in Figure 4E, compared with parental cells, the IC_50_ of HOS/ADM and 143B/ADM expressing sh-CCNL1 decreased by approximately 35% and 40%, respectively. These results revealed that CCNL1 played a significant role in the development of OS ADM-resistance.

To further validate the effect of CCNL1 in ADM resistance, we knockdown the expression of CCNL1 in HOS/ADM and 143B/ADM (Figure 4F, 4G). As shown in Figure 4H, 4I, downregulation of CCNL1 restored the sensitivity of ADM in HOS/ADM and 143B/ADM cells. Moreover, the apoptosis rate was also increased in the sh-CCNL1 group in comparison to the NC group exposing to ADM treatment (Figure 4J).

### CCNL1 promoted drug resistance of OS cells

**Figure 5:**
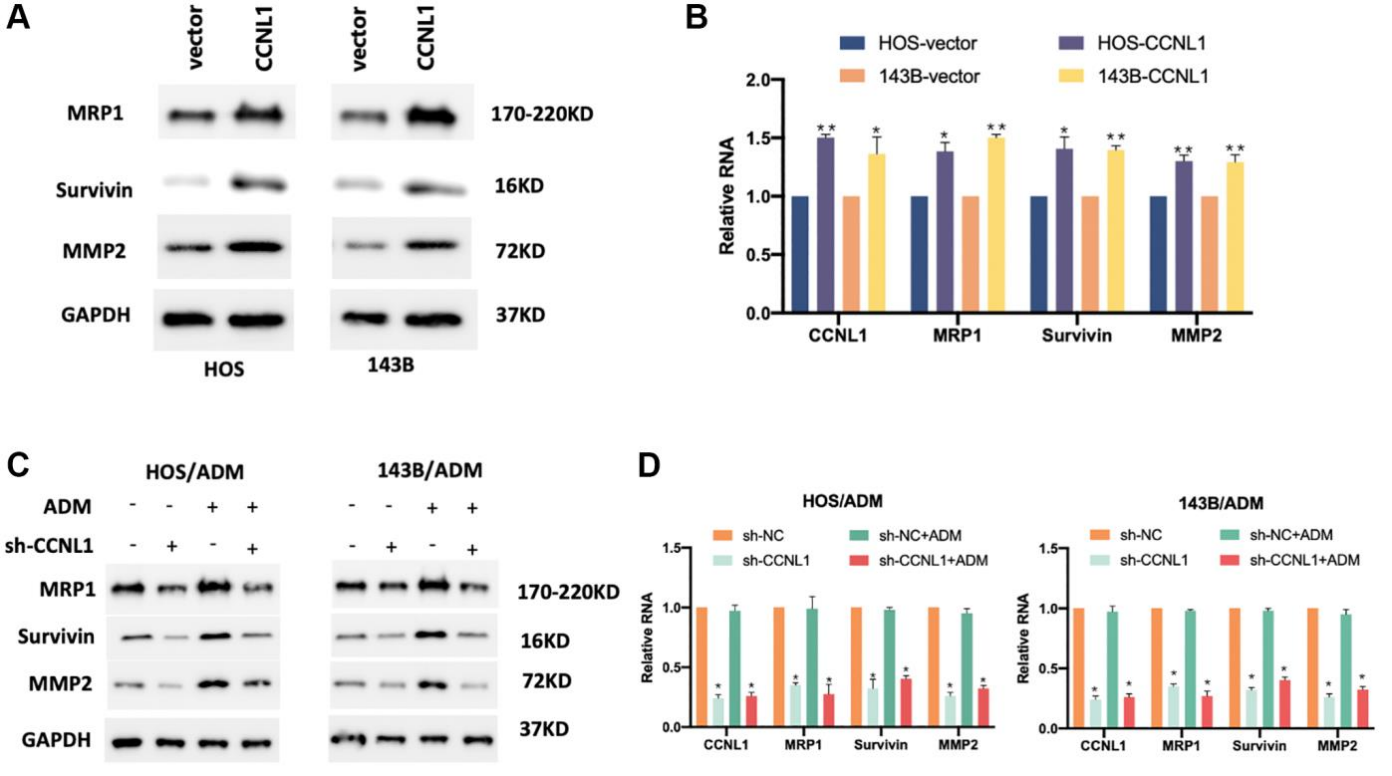
**CCNL1 promoted drug resistance of OS cells.** The expression of MPR1, Survivin, and MMP2 in HOS and 143B cells examined by western blot (**A**) and qRT-PCR analysis (**B**). ^*^P < 0.05, ^**^P < 0.01, vector vs. CCNL1. The expression of MPR1, Survivin, and MMP2 in HOS/ADM and 143B/ADM cells examined by western blot (**C**) and qRT-PCR analysis (**D**). ^*^P < 0.05, ^**^P < 0.01, sh-NC vs. sh-CCNL1.

In order to validate the effect of CCNL1 on the progression and ADM-resistant of OS cells, we explored the expression levels of tumor-related genes MRP1, Survivin, and MMP2 in CCNL1-overexpressing HOS and 143B cells. In Figure 5A, 5B, overexpression of CCNL1 could promote the expression of MRP1, Survivin, and MMP2. Moreover, knockdown of CCNL1 in HOS/ADM and 143B/ADM remarkedly reduced the MRP1, Survivin, and MMP2 (Figure 5C, 5D). The above results further verified that CCNL1 promote the drug resistance of OS cells.

### Knockdown of CCNL1 inhibits tumor growth and ADM-resistance

**Figure 6:**
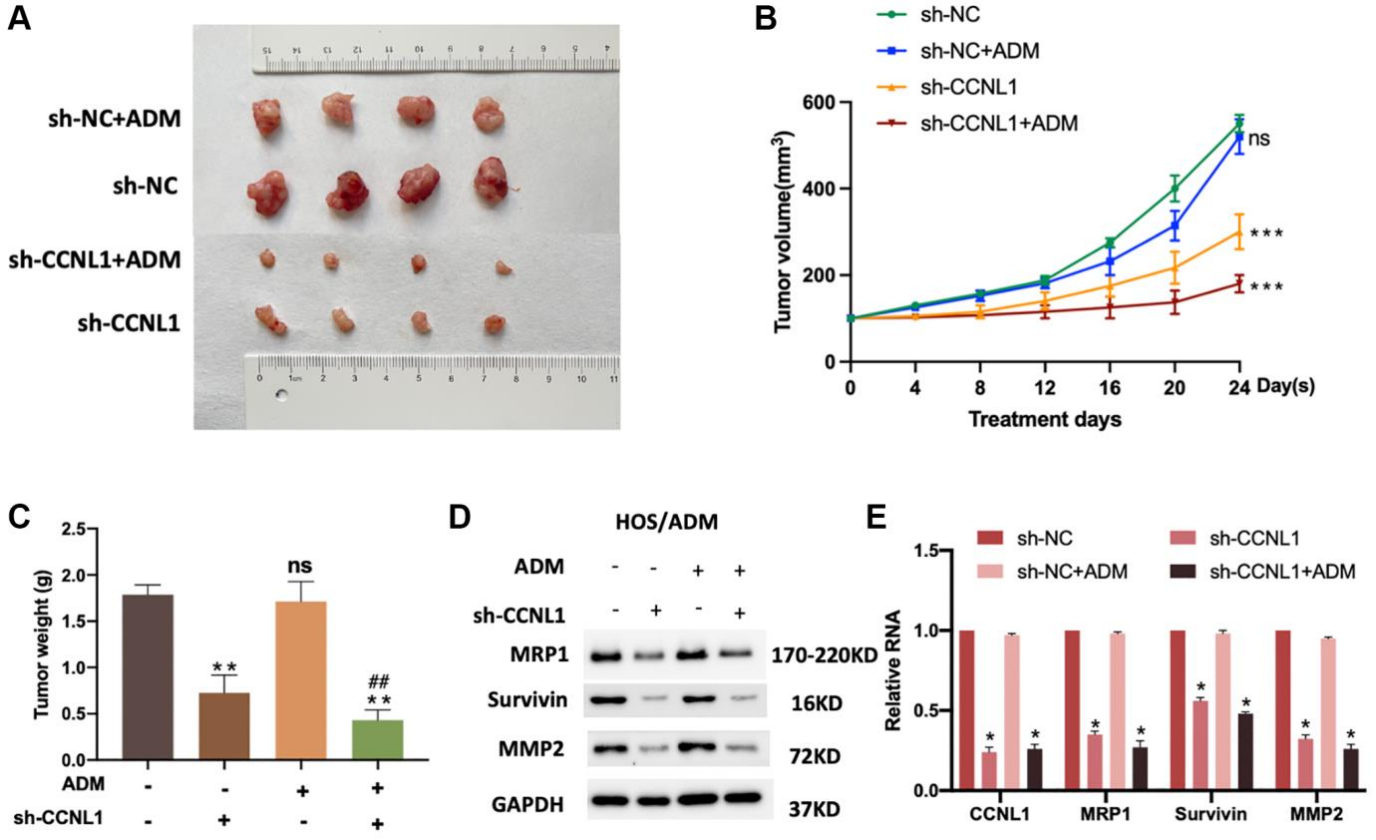
**Knockdown of CCNL1 suppresses tumor growth.** (**A**) Picture of tumors removed from NOD/SCID mice 24 days after HOS cells transfected with vector and sh-CCNL1 with or without ADM intervention. (**B**) Tumor size of HOS/ADM model every four days. ns, no significance, vs. sh-NC. ^***^P < 0.001, vs. sh-NC. (**C**) Tumor weight of the above model on the 24th day of ADM intervention or equal volumes of saline. ^**^P < 0.01, ^##^*P* < 0.01, vs. sh-CCNL1. ns, no significance. The expression of MRP1, Survivin, and MMP2 of tumors were detected by western blot (with or without exposure to ADM, knockdown, or no knockdown of CCNL1) (**D**), and qRT-PCR (**E**). ^*^P < 0.05, vs. sh-NC.

For exploring the effect of CCNL1 on the progression and chemosensitivity of OS, we conducted *in vivo* experiments. The mice were inoculated with vector-transfected or sh-CCNL1-transfected HOS/ADM cells with or without ADM intervention. As displayed in Figure 6A, 6B, knockdowning CCNL1 significantly reduced the growth rate of HOS/ADM cells with or without ADM intervention. Moreover, as shown in Figure 6C, consistent with the differences of tumor size above, the tumor weight of sh-CCNL1 group was lower than sh-NC group with or without ADM intervention. In addition, consistent with the *in vitro* results, the MRP1, Survivin and MMP2 in sh-CCNL1 group were significantly lower (Figure 6D, 6E). The above results further demonstrated that CCNL1 played a vital role in OS ADM-resistance.

### PI3K/AKT-mTOR pathway participated in the development and ADM-resistance of OS

**Figure 7:**
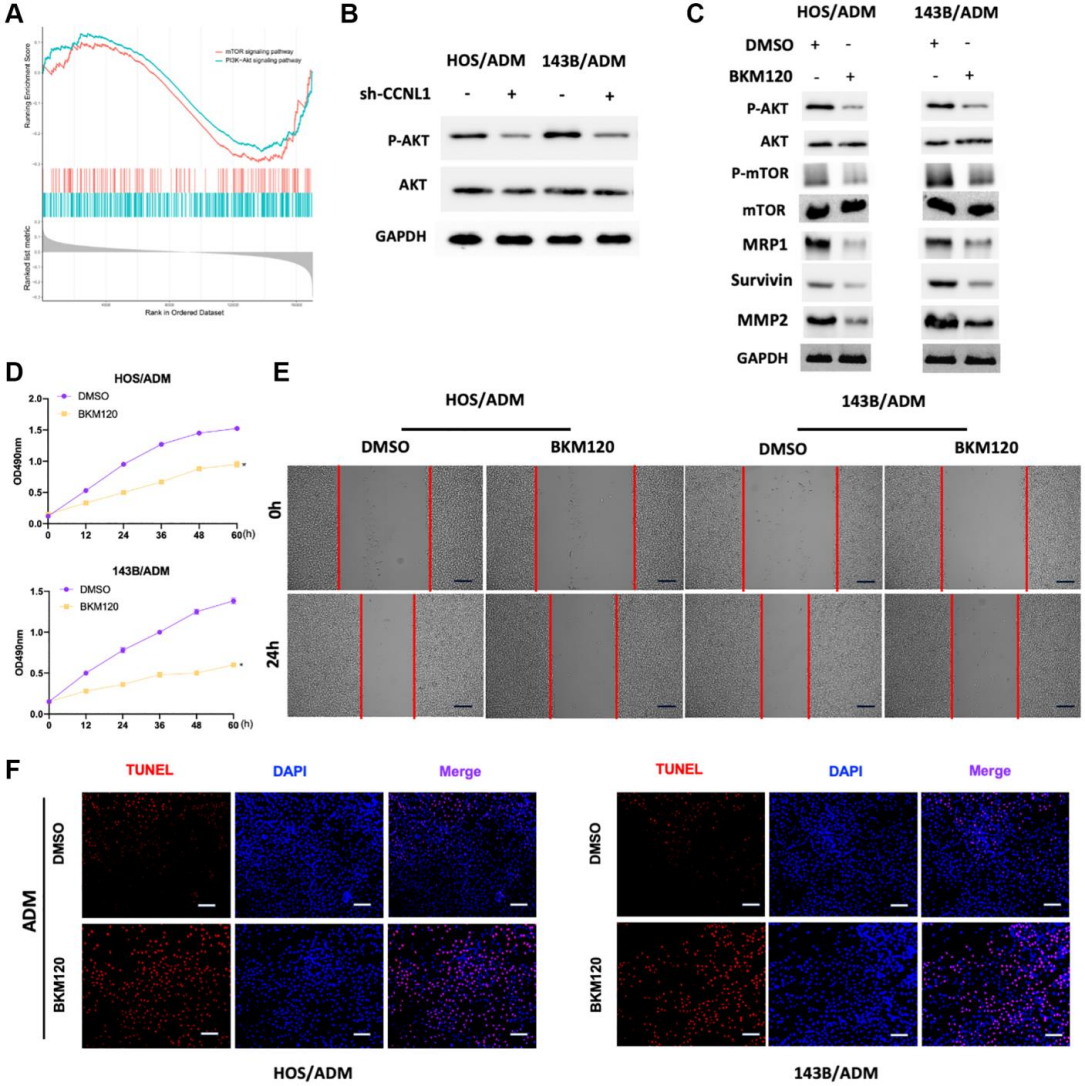
**PI3K/AKT-mTOR pathway participated in the development and ADM-resistance.** (**A**) The PI3K-Akt and mTOR were significantly enriched in OS patients with high CCNL1 levels by GSEA analysis. (**B**) Western blot results of the p-AKT and AKT in HOS/ADM and 143B/ADM cells. (**C**) Western blot results of the AKT (p-AKT), mTOR (p-mTOR), MRP1, Survivin, and MMP2 (with or without BKM120). (**D**) MTT assay results of the viability of HOS/ADM (143B/ADM) cells with BKM120. ^*^P < 0.05, vs. the DMSO group. (**E**) Wound-healing assay of HOS/ADM and 143B/ADM cells at 0 h, 24 h (with or without BKM120). Bar = 200 μm. (**F**) The rate of tunnel-positive cells increased in the BKM120 group in comparison to the DMSO group exposing to ADM, Bar = 200 μm.

For exploring the mechanisms about the CCNL1 effect on the development and chemoresistance of OS, we conducted GSEA based on the expression of CCNL1. As shown in Figure 7A, the mTOR and PI3K-Akt pathways were enriched in OS patients with high CCNL1 levels, which suggested that activation of the PI3K/AKT-mTOR pathway could be involved in the carcinogenesis of CCNL1. Therefore, we further explored the correlation between the PI3K/AKT-mTOR pathway and the effect of CCNL1 on OS. In Figure 7B, CCNL1 knockdown reduced the level of phosphorylated AKT (p-AKT). For further exploring the impact of PI3K/AKT-mTOR pathway on the progression and ADM-resistance of OS, BKM120, a pan-PI3K inhibitor, was added to HOS/ADM and 143B/ADM. As shown in Figure 7C BKM120 treatment remarkedly decreased the level of p-AKT and phosphorylated mTOR (p-mTOR). And the levels of Survivin, MMP2 and MRP1 were also downregulated by BKM120 administration (Figure 7C). Moreover, cell proliferation and migration were significantly inhibited by BKM120 (Figure 7D, 7E). Furthermore, BKM120 treatment also restored the chemosensitivity for ADM in ADM-resistance OS cells (Figure 7F).

### PI3K/AKT-mTOR pathway was related to the CCNL1-induced ADM resistance and progression of OS

**Figure 8:**
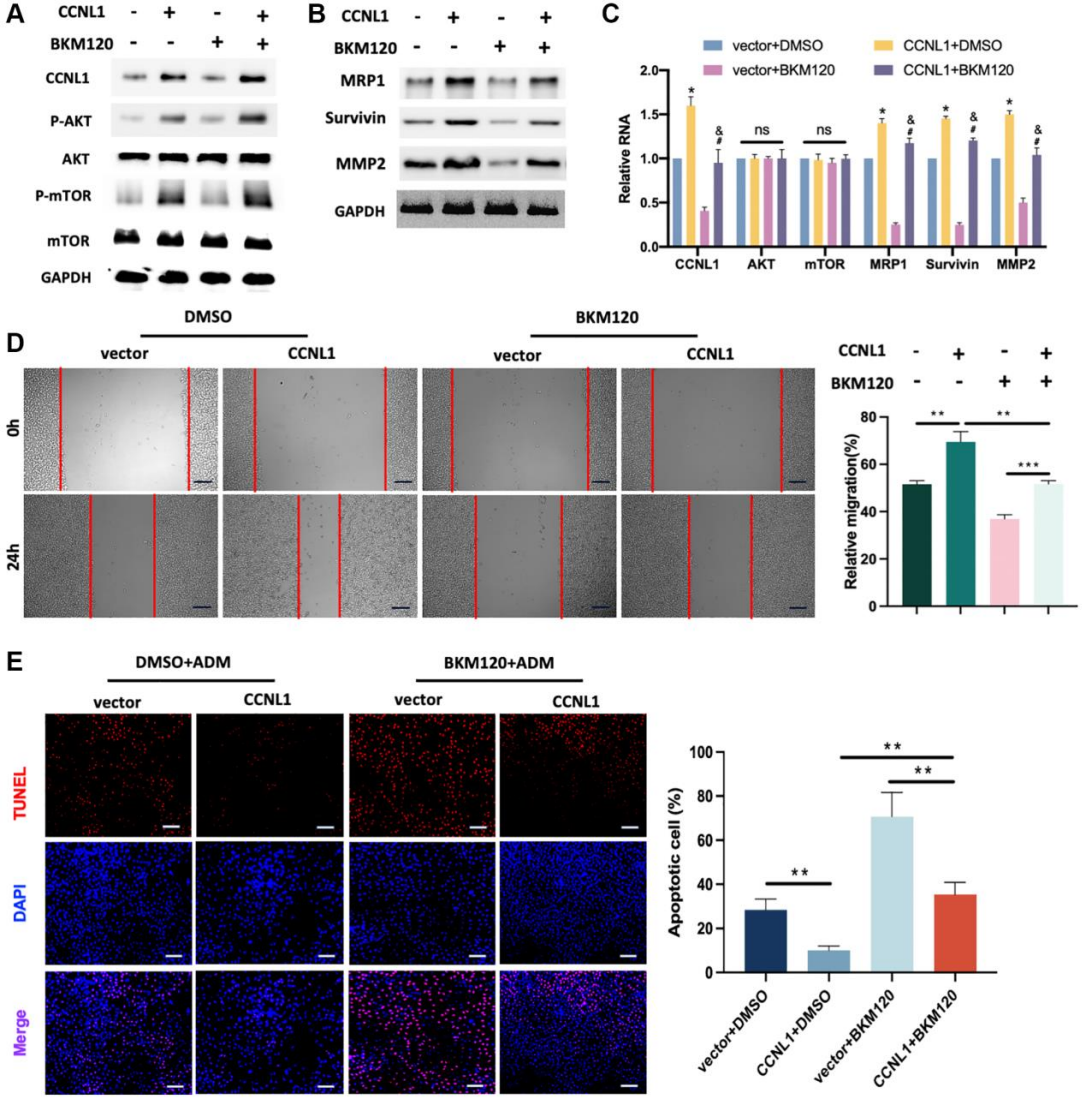
**PI3K/AKT-mTOR pathway was related to the CCNL1-induced ADM resistance and progression of OS.** (**A**) Western blot results of CCNL1, p-AKT, AKT, p-mTOR, mTOR, and GAPDH in HOS cells. (**B**) Western blot results of MRP1, Survivin, MMP2, and GAPDH in HOS cells. (**C**) qRT-PCR results of the related mRNAs CCNL1, AKT, mTOR, MRP1, Survivin, and MMP2 in HOS cells. ^*^P < 0.05, vs. vector+DMSO; ^#^*P* < 0.05, vs. CCNL1+DMSO; ^&^*P*< 0.05, vs. vector+BKM120. (**D**) Wound-healing assay of HOS cells at 0 h, 24 h. Bar = 200 μm. ^*^P < 0.05, ^**^P < 0.01. (**E**) The rate of tunnel-positive HOS cells when exposing to ADM. Bar = 200 μm. ^**^P < 0.01.

To further confirm whether CCNL1 regulates the ADM drug resistance and tumorigenicity of OS through the PI3K/AKT-mTOR pathway, we conducted a series of rescue experiments. In Figure 8A, overexpression of CCNL1 enhanced the expression of p-AKT and p-mTOR, which could be partially inhibited by BKM120. Moreover, BKM120 reversed the upregulation of MRP1, Survivin, and MMP2 by CCNL1-overexpression (Figure 8B, 8C). The results of wound-healing assay suggested that the effect of CCNL1 on promoting migration could be partially inhibited by BKM120 (Figure 8D). In addition, as shown in Figure 8E, CCNL1-overexpression induced ADM-resistance was partially reversed by BKM120 treatment. In conclusion, the above results indicated that CCNL1 promoted the ADM-resistance and tumorigenicity of OS via the PI3K/AKT-mTOR pathway.

## DISCUSSION

ADM, DDP and MTX are three chemotherapy drugs commonly used in the clinical treatment of OS [14–16]. However, the resistance to these drugs seriously threatens the overall survival rate of OS patients, which is still a major obstacle to the current clinical treatment of OS [17, 18]. In this study, we firstly demonstrate that CCNL1 is highly expressed in OS and over-expressed in low-Huvos grade (1–2) OS patients, suggesting that CCNL1 may be a promising target for chemoresistance of OS.

It has been reported that CCNL1 is associated with the development of various tumors, such as HNSCC [8, 19], Ewin’s sarcoma [20], and prostate cancer [9]. However, the relationship between CCNL1 and OS remains unclear. In this study, we have revealed that CCNL1 is able to promote the ADM-resistance and tumorigenicity of the OS. From a biological point of view, CCNL1 is involved in the regulation of OS tumorigenesis and chemotherapy resistance, providing new clues to the mechanism of ADM-resistance and a new biological therapeutic target for improving the treatment of ADM-resistant OS.

Interestingly, several studies have reported that CCNL1 is directly regulated by miR-5195-3p and miR-199-5p [9, 20]. Currently, miRNAs-loaded extracellular vesicles or biomaterials are considered to be an efficient means of bioregulation [21]. Therefore, we speculate that the delivery of CCNL1-inhibiting miRNAs to ADM-resistant OS cells through extracellular vesicles or biomaterials maybe a promising approach to solve the problem of drug resistance. However, the choice of which cell-derived vesicles or suitable biomaterials remains to be further studied.

As a classic biological signaling pathways in the human body, the PI3K/AKT-mTOR pathway controls a variety of important cell functions such as transcription, translation, and proliferation, and its dysfunction may lead to various human cancers [22]. It has been previously reported that PI3K/AKT-mTOR pathway is involved in the drug resistance process [23–26]. The regulatory relationship between CCNL1 and the PI3K/AKT-mTOR pathway is important for elucidating the mechanism of CCNL1-mediated OS ADM resistance. In this study, we have demonstrated that the PI3K/AKT-mTOR pathway is involved in CCNL1-mediated OS ADM resistance. However, during this biological regulation process, CCNL1 may interact with other biological proteins or small molecules, which is a limitation of this study and needs further exploration.

In conclusion, in this study, we explored and elucidated its role in drug resistance in OS starting from CCNL1. Compared with other oncogenes, CCNL1 has its particularity and is quite active in the occurrence of many tumors, which may be a key node in the occurrence of tumor drug resistance. This study will play a guiding or supplementary role in the treatment and research of drug resistance in OS, including some other tumors.

## Supplementary Materials

Supplementary Figures

Supplementary Table 1
