# Supplementary material for: Cyclin L1 participates in Adriamycin resistance and progression of osteosarcoma via PI3K/AKT-mTOR pathway
Source: Aging (Albany NY). 2024 Jun 26;16(14):11208–23. doi: 10.18632/aging.205972 (PMC11315378; doi:10.18632/aging.205972)
Supplement: Supplementary Figures [file aging-16-205972-s001.pdf]

SUPPLEMENTARY FIGURES

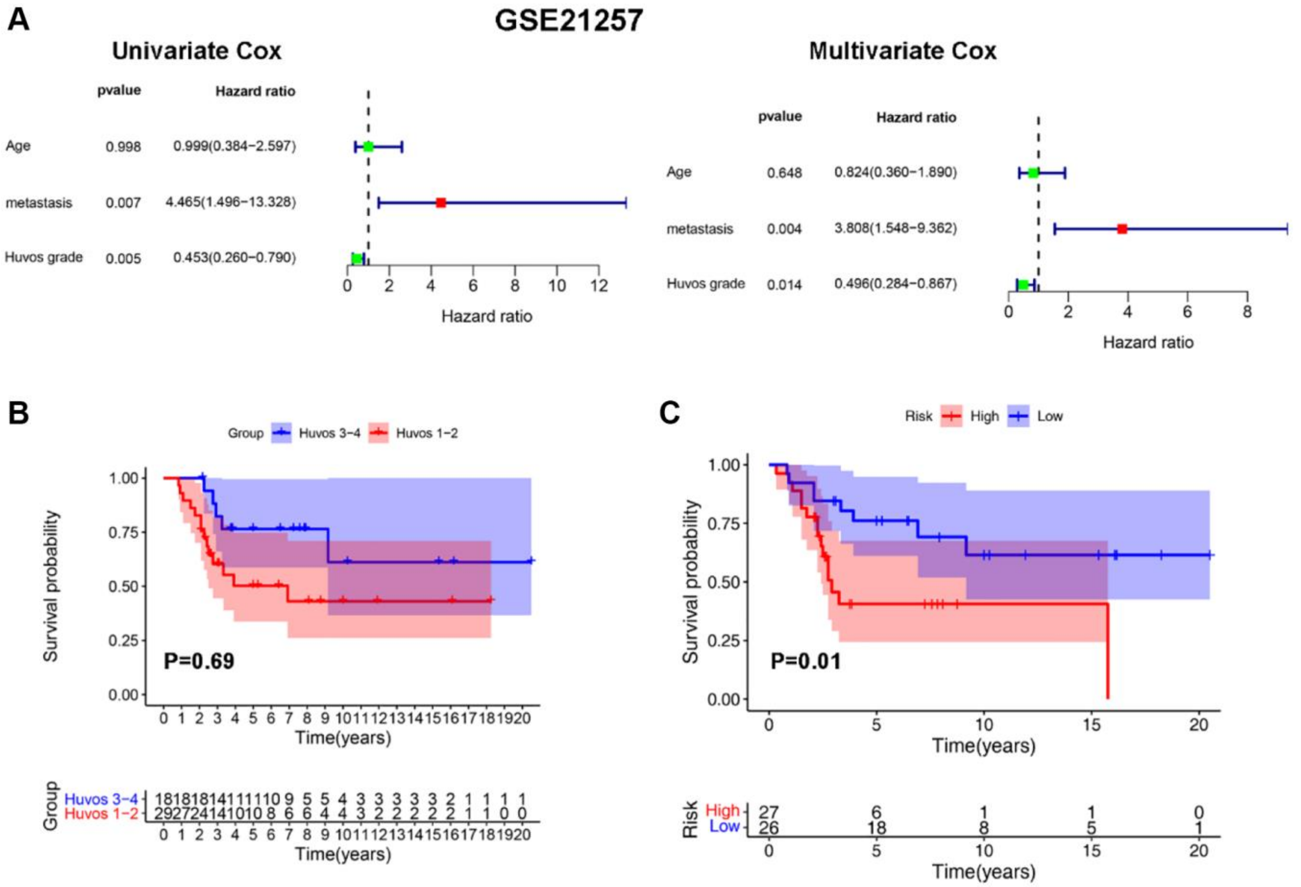

Supplementary Figure 1. The prognostic value of Huvos grade and CCNL1 in GSE21257 OS cohort. The Cox regression analysis of Huvos grade in GSE21257 (**A**). The overall survival analysis between Huvos 1–2 and Huvos 3–4 in GSE21257 (**B**). High CCNL1 level was correlated with worse prognosis in OS in GSE21257 (**C**).

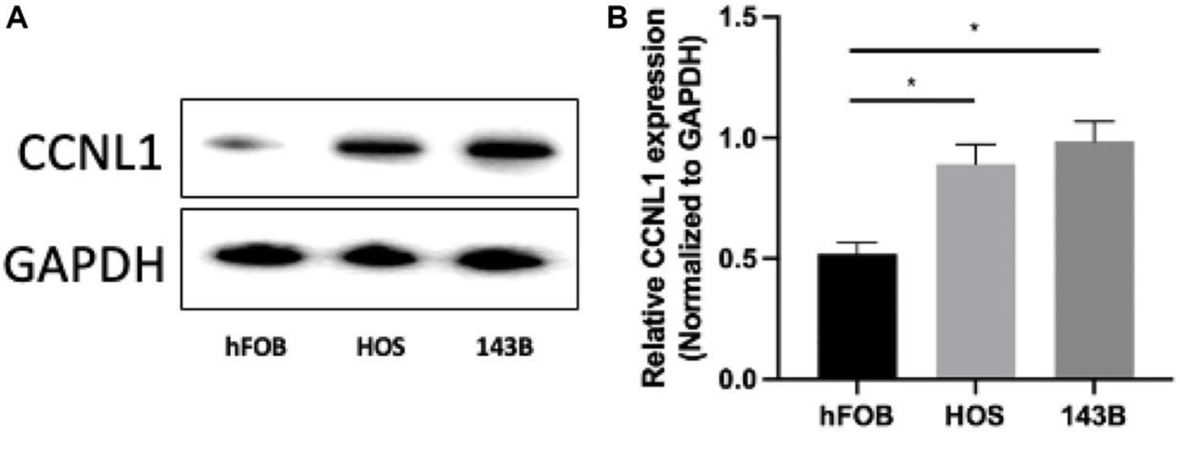

Supplementary Figure 2. High expression of CCNL1 in OS cells. The expression level of CCNL1 in hFOB, HOS and 143B was examined by western blot (**A**) and qRT-PCR (**B**). *$P < 0.05$ vs. hFOB.
