# Supplementary material for: Cyclin L1 participates in Adriamycin resistance and progression of osteosarcoma via PI3K/AKT-mTOR pathway
Source: Aging (Albany NY). 2024 Jun 26;16(14):11208–23. doi: 10.18632/aging.205972 (PMC11315378; doi:10.18632/aging.205972)
Supplement: Supplementary Table 1 [file aging-16-205972-s002.pdf]

SUPPLEMENTARY TABLE

Supplementary Table 1. The primers used for PCR.

Gene (human)	Forward Primer (5′–3′)	Reverse Primer (3′–5′)
MRP1	TGCCTGTTTTGGTAAAGAACTG	CTTGGAGGAGTACACAACCTTC
P-gp	TCTATGGTTGGCAACTAACACT	CTCCTGAGTCAAAGAAACAACG
CCNL1	CGTCAAACACAGTTTCGAGATT	GTCCTTTTTCCTCTTAACTGGC
Survivin	CCGCATCTCTACATTCAAGAAC	CTCCTTGAAGCAGAAGAAACAC
MMP2	ATTGTATTTGATGGCATCGCTC	ATTCATTCCCTGCAAAGAACAC
AKT	TGACCATGAACGAGTTTGAGTA	GAGGATCTTCATGGCGTAGTAG
mTOR	GAGATACGCTGTCATCCCTTTA	CTGTATTATTGACGGCATGCTC
GAPDH	GTCTCCTCTGACTTCAACAGCG	ACCACCCTGTTGCTGTAGCCAA
